# Identification of Sox2 and NeuN Double-Positive Cells in the Mouse Hypothalamic Arcuate Nucleus and Their Reduction in Number With Aging

**DOI:** 10.3389/fnagi.2020.609911

**Published:** 2021-03-11

**Authors:** Ayumu Sugiura, Tatsuhiro Shimizu, Takeshi Kameyama, Tomohiko Maruo, Shin Kedashiro, Muneaki Miyata, Kiyohito Mizutani, Yoshimi Takai

**Affiliations:** Division of Pathogenetic Signaling, Department of Biochemistry and Molecular Biology, Kobe University Graduate School of Medicine, Kobe, Japan

**Keywords:** aging, arcuate nucleus, hypothalamic neuronal cell, Sox2, NeuN

## Abstract

The hypothalamus plays a central role in homeostasis and aging. The hypothalamic arcuate nucleus (ARC) controls homeostasis of food intake and energy expenditure and retains adult neural stem cells (NSCs)/progenitor cells. Aging induces the loss of NSCs and the enhancement of inflammation, including the activation of glial cells in the ARC, but aging-associated alterations of the hypothalamic cells remain obscure. Here, we identified Sox2 and NeuN double-positive cells in a subpopulation of cells in the mouse ARC. These cells were reduced in number with aging, although NeuN-positive neuronal cells were unaltered in the total number. Diet-induced obesity mice fed with high-fat diet presented a similar hypothalamic alteration to aged mice. This study provides a new insight into aging-induced changes in the hypothalamus.

## Introduction

Aging is one of the biggest issues that human beings have been tackling. Studies using various model organisms from yeast, a unicellular eukaryote, to rhesus monkey, a primate, have characterized aging, including genome instability, telomere shortage, mitochondrial dysfunction, and so on (López-Otín et al., 2013). However, these are valid observations with aging, and our understanding of the principle of aging mechanisms is still poor. The extension of a healthy life span is an important subject for both natural science and economy. Despite numerous studies, it appears that only caloric restriction succeeds in extending the life span of model organisms and humans (Klass, 1977; Weindruch et al., 1986; Chippindale et al., 1993; Lin et al., 2000; Colman et al., 2009). Caloric restriction extends the life span of rats (McCay et al., 1935), whereas excess calorie intake increases the risk for diseases and consequently shortens the life span (Biliński et al., 2015). Thus, controlling feeding can be a point to controlling life span. Recent studies have revealed that signaling pathways, such as the insulin-like growth factor pathway, are associated with the effects of caloric restriction (Lakowski and Hekimi, 1998; Vitale et al., 2019), but it is largely unknown how caloric restriction delays natural aging or induces the hormesis effect.

The hypothalamus is a part of the midbrain and is composed of various functional nuclei. The arcuate nucleus (ARC) is a nucleus located in the mediobasal hypothalamus and plays a central role in homeostasis, including appetite, energy expenditure, emotion, and sleep. Neuronal cells in the ARC control homeostasis in response to the hormonal/peptide signals released from peripheral organs and tissues. Food intake and energy expenditure are mainly regulated by two types of neuronal cells in the ARC, pro-opiomelanocortin (POMC)-expressing neuronal cells and neuropeptide Y (NPY)/agouti-related protein (AgRP)-co-expressing neuronal cells (Cowley et al., 2001; Williams and Elmquist, 2012). POMC is cleaved into multiple peptide hormones, including adrenocorticotropic hormone (ACTH) and α-melanocyte-stimulating hormone (α-MSH; Harno et al., 2018). The neuronal cells expressing POMC or NPY receive signal hormones/peptides secreted from peripheral organs and tissues and prompt to take an adequate action by activating the brain neuronal circuit (Hâkansson et al., 1998). In a fasted state, ghrelin released from the stomach activates the NPY/AgRP neuronal cells, which promote feeding behavior. In a fed state, leptin released from the adipose tissue inhibits the NPY/AgRP neuronal cells and activates the POMC neuronal cells, thereby suppressing feeding behavior and increasing energy expenditure (Kim et al., 2018).

The ARC is also known as a region where adult neurogenesis occurs (Kokoeva et al., 2005). Neural stem cells (NSCs) characterized by the ability to self-renew and differentiate into neuronal cells or glial cells are labeled with markers, including Sox2, Bmi1, and Nestin (Lendahl et al., 1990; Molofsky et al., 2003; Favaro et al., 2009; Zhang and Jiao, 2015). Although defining the locations of hypothalamic NSCs and progenitor cells is still under debate, tanycytes, the cells aligning the wall of the third ventricle (3V), are considered NSCs in the hypothalamus (Kokoeva et al., 2005; Lee et al., 2012; Robins et al., 2013). Tanycytes, termed by their stretched shape (Horstmann, 1954), act as the NSCs in response to signals in the cerebrospinal fluid (CSF; Pierce and Xu, 2010; Li et al., 2012; McNay et al., 2012; Haan et al., 2013; Chaker et al., 2016; Klein et al., 2019). In addition to the role of NSCs, tanycytes are involved in the regulation of energy expenditure and calorie intake (Clasadonte and Prevot, 2018). Tanycytes sense and uptake nutrients and signaling molecules from the CSF in the 3V and convey metabolic signals to the neuronal cells in the parenchyma through their stretched projections (Elizondo-Vega et al., 2015; Prevot et al., 2018).

Increased inflammation and loss of NSCs are observed in the hypothalamus of aged mice and high-fat diet (HFD)-fed mice (Li et al., 2012; Zhang et al., 2013, 2017), supporting excess calorie intake, which in turn accelerates aging and its common machinery, leading to hypothalamic dysregulation. Consistently, mice that genetically suppress inflammation or that are implanted with young stem cells to the hypothalamus display a delay in aging and a longer life span (Li et al., 2012; Zhang et al., 2013, 2017). Thus, alterations of NSCs, such as decline in self-renew and/or neuronal or glial differentiation, may be involved in individual aging processes, but their molecular mechanisms remain elusive.

Sox2 is a transcription factor well-known as a stem cell marker that regulates self-renewal and differentiation. *Sox2*-deficient mice experienced embryonic lethality (Avilion et al., 2003). It is widely accepted that the expression of Sox2 is reduced gradually during the differentiation of NSCs/progenitor cells to functionally mature neuronal cells and glial cells (Cavallaro et al., 2008). Accumulated evidence demonstrates that Sox2 is also expressed in a part of differentiated neuronal cells and glial cells in some regions of the brain and regulates their cellular functions (Chou et al., 2013; Cheng et al., 2019; Mercurio et al., 2019a,b). Thus, Sox2 regulates various cellular functions, suggesting its involvement in homeostasis. Here, we investigated the Sox2-positive cells in the ARC of the mouse hypothalamus to understand its role in aging and identified Sox2 and NeuN double-positive cells in a subpopulation of cells in the ARC of the hypothalamus.

## Materials and Methods

### Animals

C57BL/6J male mice purchased from CLEA Japan (Tokyo, Japan) were maintained under a 12/12 light-dark cycle. For a HFD experiment, 10-week-old male mice were fed with HFD (45% fat of total calorie) and control diet (CD; 10% fat of total calorie) (HD12451 and D12450H, respectively, Research Diet, New Brunswick, NJ, USA) *ad libitum* for 16 weeks. All animal experiments were performed in accordance with the guidelines of the institution and approved by the administrative panel on laboratory animal care of Kobe University. This study was approved by the president of Kobe University after being reviewed by the Animal Care and Use Committee of Kobe University (approval number: 30–27), and animal experiments were conducted in accordance with the regulations for animal experimentation of Kobe University.

### Immunofluorescence Microscopy

Deeply anesthetized mice were transcardially perfused with an ice-cold phosphate buffer (PB; pH 7.4) containing a fixative composed of 2% paraformaldehyde, 4% sucrose, 1 mM sodium pyruvate, Hanks' balanced salt solution containing 1 mM CaCl_2_ and 1 mM MgCl_2_ (Thermo Fisher Scientific, Waltham, MA, USA), 3 U/ml heparin sodium, and 10 mM HEPES (pH 7.3). The brains were dissected and incubated in the same fixative at 4°C for 4 h and then dehydrated in 100 mM PB containing 20% sucrose for 2 h, followed by incubation in 100 mM PB containing 25% sucrose (pH7.4) overnight. The brains were placed in Tissue-Tek O.C.T. Compound (Sakura Finetek, Tokyo, Japan) and frozen in acetone chilled with dry ice. The sections of 12-μm thickness were mounted on glass slides and incubated in HistoVT One, an antigen retrieval solution (Nacalai Tesque, Kyoto, Japan), at 62°C for 20 min, and then with Blocking One Histo (Nacalai Tesque) at room temperature for 20 min. The specimens were incubated with primary antibodies (Abs) in Can Get Signal Immunoreaction Enhancer Solution B (Toyobo, Osaka, Japan) at 4°C overnight. After three to four washes of 10 min each in phosphate-buffered saline (PBS), the samples were incubated with secondary Abs and 1 μg/ml 4',6-diamidino-2-phenylindole (DAPI; Nacalai Tesque) in the PBS at room temperature for 2 h. After three to four washes of 10 min each in PBS, the samples were mounted in FluorSave reagent (Merck Millipore, Billerica, MA, USA). Images were captured with a BZ-X710 (Keyence, Osaka, Japan) fluorescence microscope. Maximum signal intensity projection images were created from ~15 images collected at a 500 nm step along the z-axis. The number of cells with each marker in the ARC was counted manually, and the average number of cells of two to four fields was presented. Each Ab was obtained as follows: Sox2 (sc-17320, Y-17, Santa Cruz Biotechnology, Dallas, TX, USA), Nestin (AF2736, R&D Systems, Minneapolis, NE, USA), NeuN (MAB377X, Merck Millipore), glial fibrillary acidic protein (GFAP; ab134436, abcam, Cambridge, UK), PDGFRα (558774, BD Pharmingen, Franklin Lakes, NJ, USA), Iba1 (019-19741, Wako, Osaka, Japan), ACTH (sc-57021, B427, Santa Cruz Biotechnology), and AgRP (AF634, R&D Systems). Alexa Fluor 488-conjugated Sox2 Ab was obtained from Thermo Fisher Scientific (#53-9811-82, Thermo Fisher Scientific).

### Statistical Analysis

The number of counted cells was indicated as dot plots. The value of *p* of each analysis was calculated as a two-tailed unpaired *t*-test and is shown in figures. Statistical analysis was performed with GraphPad Prism 8 software (GraphPad Software, San Diego, CA, USA).

## Results

### Aging-Associated Reduction of Sox2-Positive Cells in Number in the ARC

**Figure 1:**
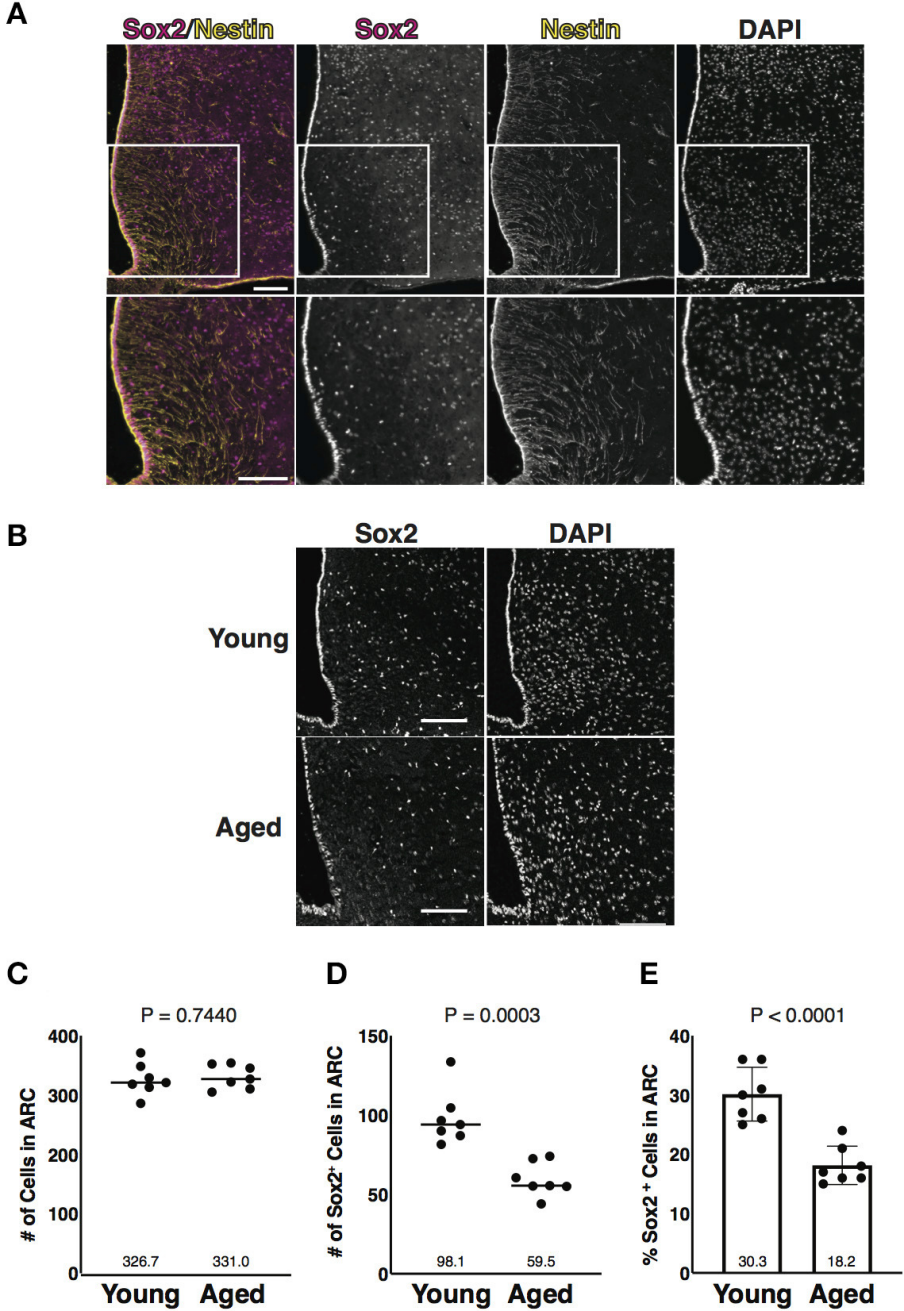
Aging-associated reduction of Sox2-positive cells in number in the arcuate nucleus (ARC). **(A)** Representative images of the immunofluorescence labeling of Sox2 and Nestin and nuclear labeling with 4',6-diamidino-2-phenylindole (DAPI) on the coronal sections of the hypothalamus obtained from 3-month-old male mice. Insets of upper images are shown in bottom images. Scale bars, 100 μm. **(B)** Representative images of the immunofluorescence labeling of Sox2 and nuclear labeling with DAPI on the coronal sections of the hypothalamus obtained from 3-month-old (young) and 25-month-old (aged) male mice. Scale bars, 100 μm. **(C,D)** Dot plots represent the number of all cells and Sox2-positive cells: **(C)** total number of cells counted by DAPI and **(D)** the number of Sox2-positive cells. **(E)** Bar graph represents the ratio of Sox2-positive cells to all cells. The mean of each condition is indicated on graph and shown as horizontal lines. Error bars, SEM. Young mice were 3-month-old. Aged mice were 24 to 26-month-old.

A transcription factor Sox2, one of the several markers for NSCs, regulates various signaling pathways and the maintenance of cell stemness (Schaefer and Lengerke, 2020). Tanycytes are Nestin and Sox2 double-positive cells and are widely accepted as the NSCs in the ARC (Lee et al., 2012; Li et al., 2012; Robins et al., 2013). Sox2-positive cells were previously found in the parenchyma of the ARC, but they have not been investigated in this region (Li et al., 2012). Consistent with these previous observations, we first identified Nestin and Sox2 double-positive cells along the wall of the 3V, which corresponded to tanycytes, and Nestin-negative Sox2-positive cells in the parenchyma of the ARC (Figure 1A). To understand hypothalamic aging, we focused on Sox2-positive cells in the parenchyma of the ARC. Frozen thin sections from young (3-month-old) and aged (24 to 26-month-old) mice were immunostained with the anti-Sox2 Ab (Figure 1B). The cells estimated by nuclear staining with DAPI in the ARC were unaltered in total number with aging (Figure 1C), but Sox2-positive cells were significantly reduced in number in aged mice (Figures 1D,E). These results suggest that Sox2-positive cells in the parenchyma of the ARC are reduced in number with aging as described for the tanycytes (Zhang et al., 2017).

### Identification of Sox2 and NeuN Double-Positive Cells in the ARC

**Figure 2:**
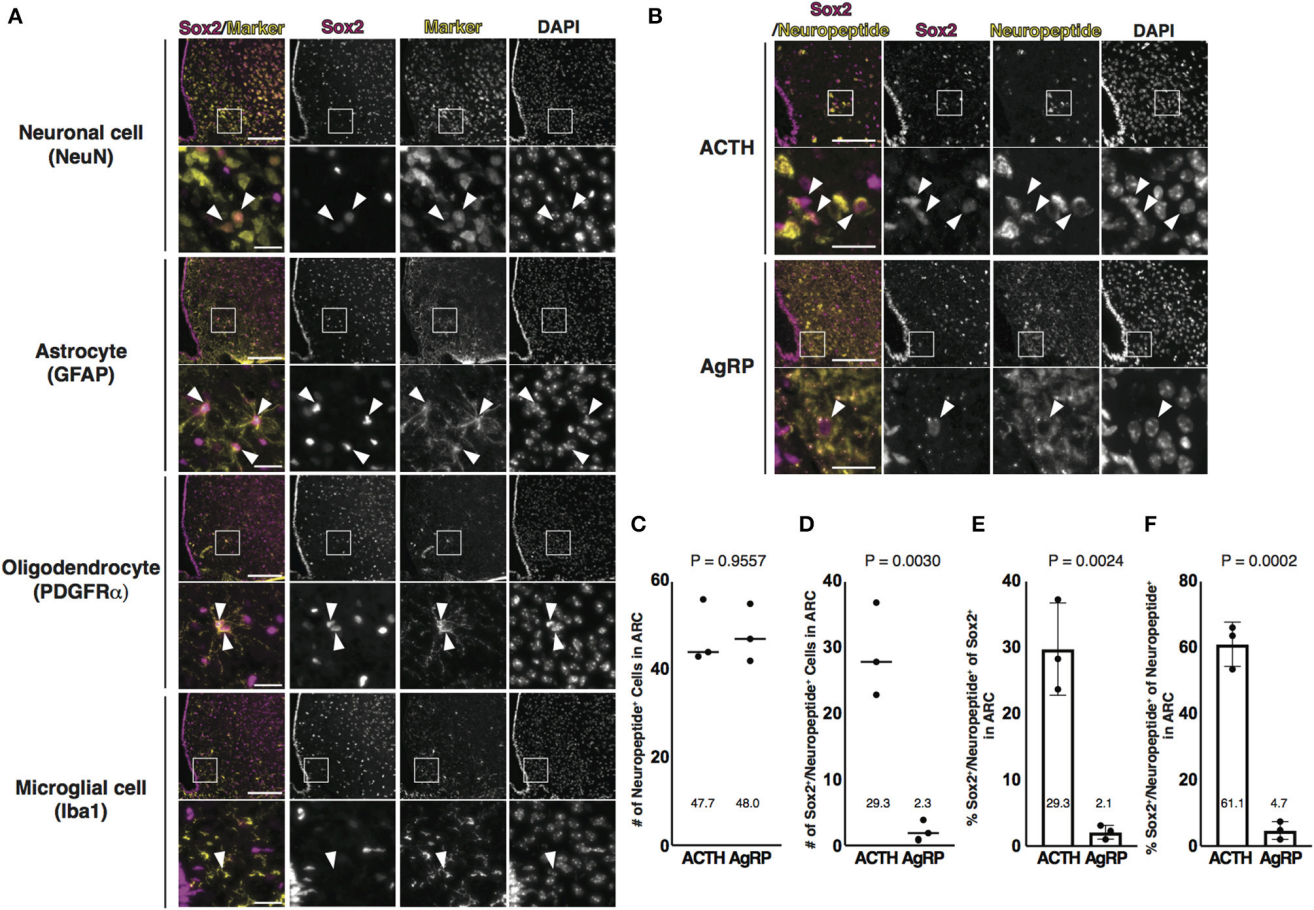
Identification of Sox2 and NeuN double-positive cells in the ARC. **(A,B)** Representative images of the immunofluorescence labeling of indicated Abs and nuclear labeling with DAPI on the coronal sections of the ARC from 3-month-old male mice: **(A)** Arrowheads indicate cells labeled with each marker; **(B)** Arrowheads indicate Sox2 and neuropeptide double-positive cells. Scale bars in low magnification images, 100 μm. Scale bars in high magnification images, 20 μm. **(C,D)** Dot plots represent the number of neuropeptide-positive cells and Sox2 and neuropeptide double-positive cells: **(C)** neuropeptide-positive cells and **(D)** Sox2 and neuropeptide double-positive cells. The mean of each condition is indicated on graphs and shown as horizontal lines. **(E,F)** Bar graphs represent the ratio of Sox2 and neuropeptide double-positive cells to Sox2-positive and neuropeptide-positive cells: **(E)** Sox2-positive cells and **(F)** neuropeptide-positive cells. Error bars, SEM.

Next, we attempted to characterize Sox2-positive cells in the parenchyma of the ARC. The ARC consists of NSCs, neuronal cells, and glial cells, such as astrocytes, oligodendrocytes, and microglial cells. Neuronal cells, astrocytes, and oligodendrocytes are differentiated from radial glial cells expressing Sox2, whereas microglial cells originate from erythromyeloid precursors (Kierdorf et al., 2013). Frozen thin sections prepared from 3-month-old mice were co-stained with Sox2 and respective markers for each cell type: NeuN for neuronal cells; GFAP for astrocytes; platelet-derived growth factor receptor α (PDGFRα) for oligodendrocytes; and ionized calcium-binding adapter molecule 1 (Iba1) for microglial cells. A part of neuronal cells, astrocytes, and oligodendrocytes was co-labeled with Sox2, although astrocytes and oligodendrocytes were fewer than neuronal cells (Figure 2A). Microglial cells were not found to be labeled with Sox2 in this study. Of neuronal cells, Sox2 was expressed in a part of ACTH-positive cells but rarely in AgRP-positive cells (Figures 2B–F). These results indicate that Sox2-positive cells in the parenchyma of the ARC are not only NSCs but also neuronal cells, astrocytes, and oligodendrocytes and that Sox2 and NeuN double-positive cells are much more than Sox2 and GFAP double-positive and Sox2 and PDGFRα double-positive cells in the ARC.

### Aging-Associated Reduction of Sox2 and NeuN Double-Positive Cells in Number in the ARC

**Figure 3:**
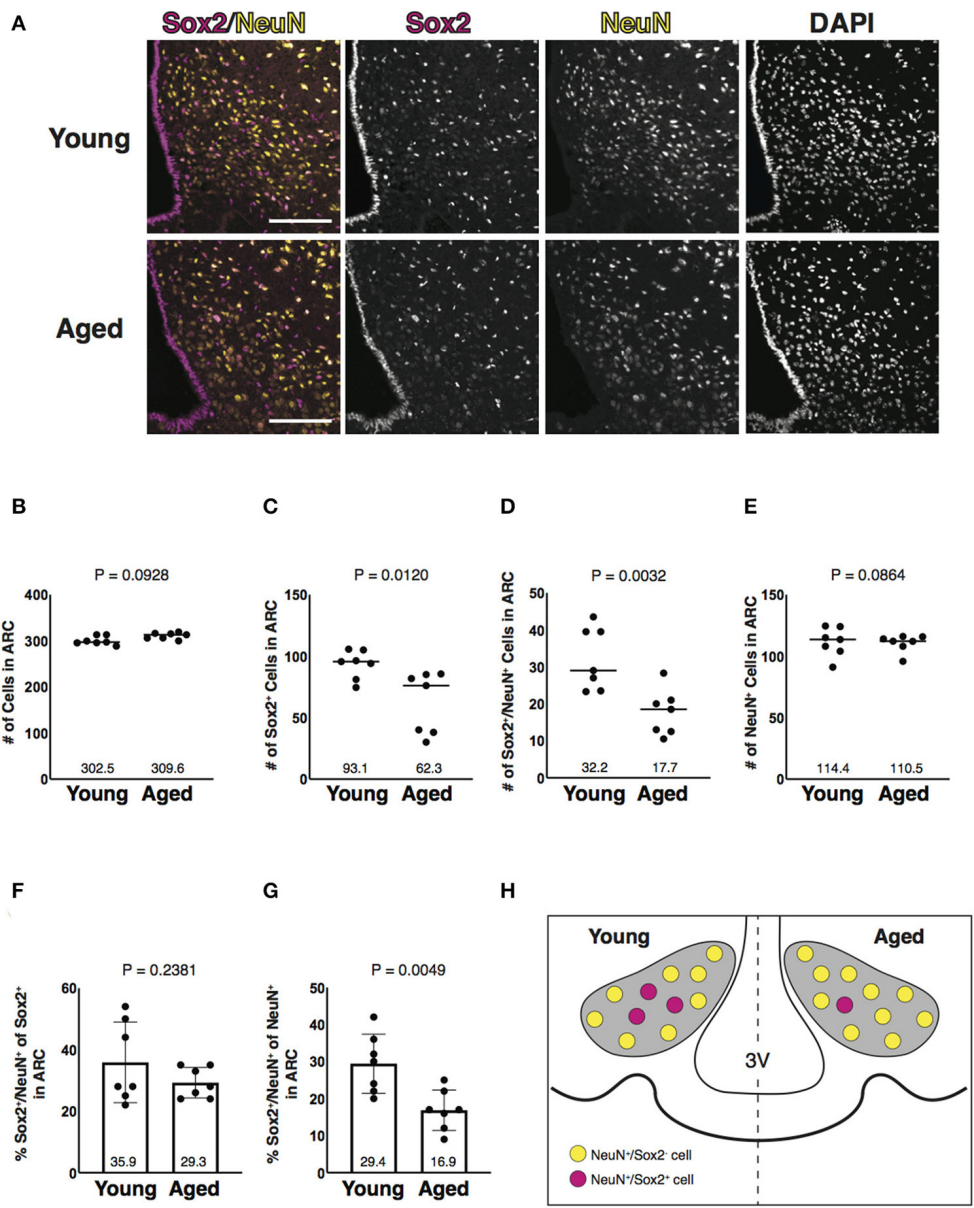
Aging-associated reduction of Sox2 and NeuN double-positive cells in number in the ARC. **(A)** Representative images of the immunofluorescence labeling of Sox2 and NeuN and nuclear labeling with DAPI on the coronal sections of the ARC from 3-month-old (young) and 25-month-old (aged) male mice. Scale bars, 100 μm. **(B–E)** Reduction of Sox2 and NeuN double-positive, but not NeuN-positive cells, in number in aged ARC. Dot plots represent the number of all, Sox2-positive, Sox2 and NeuN double-positive, and NeuN-positive cells: **(B)** total number of cells counted by DAPI, **(C)** Sox2-positive cells, **(D)** Sox2 and NeuN double-positive cells, and **(E)** NeuN-positive cells. The mean of each condition is indicated on graphs and shown as horizontal lines. **(F,G)** Reduction of the ratio of Sox2 and NeuN double-positive cells to NeuN-positive cells, but not to Sox2-positive cells, in number in aged ARC. Bar graphs represent the ratio of Sox2 and NeuN double-positive cells to Sox2-positive and NeuN-positive cells: **(F)** Sox2-positive cells; and **(G)** NeuN-positive cells. Error bars, SEM. Young mice were 3-month-old. Aged mice were 24 to 26-month-old. **(H)** Schematic representation of the aging-associated reduction of Sox2 and NeuN double-positive cells in number. Gray areas represent the ARC. 3V, the third ventricle.

Since NeuN-positive cells were dominantly co-labeled with Sox2 of various cells in the parenchyma of the ARC (Figure 2A), we further analyzed Sox2 and NeuN double-positive cells in aged mice (Figure 3A). Quantitative analysis revealed that Sox2 and NeuN double-positive cells were reduced in number in aged mice (Figures 3B–D). Importantly, NeuN-positive cells were unaltered in number with aging (Figure 3E). In ratiometric analysis, the fraction of Sox2 and NeuN double-positive cells in Sox2-positive cells was not altered (Figure 3F), whereas that in NeuN-positive cells was significantly reduced with aging (Figure 3G). These results indicate that aging reduces Sox2 and NeuN double-positive cells in number without affecting NeuN-positive cells in the parenchyma of the ARC (Figure 3H).

### Diet-Induced Obesity-Associated Reduction of Sox2 and NeuN Double-Positive Cells in Number in the ARC

**Figure 4:**
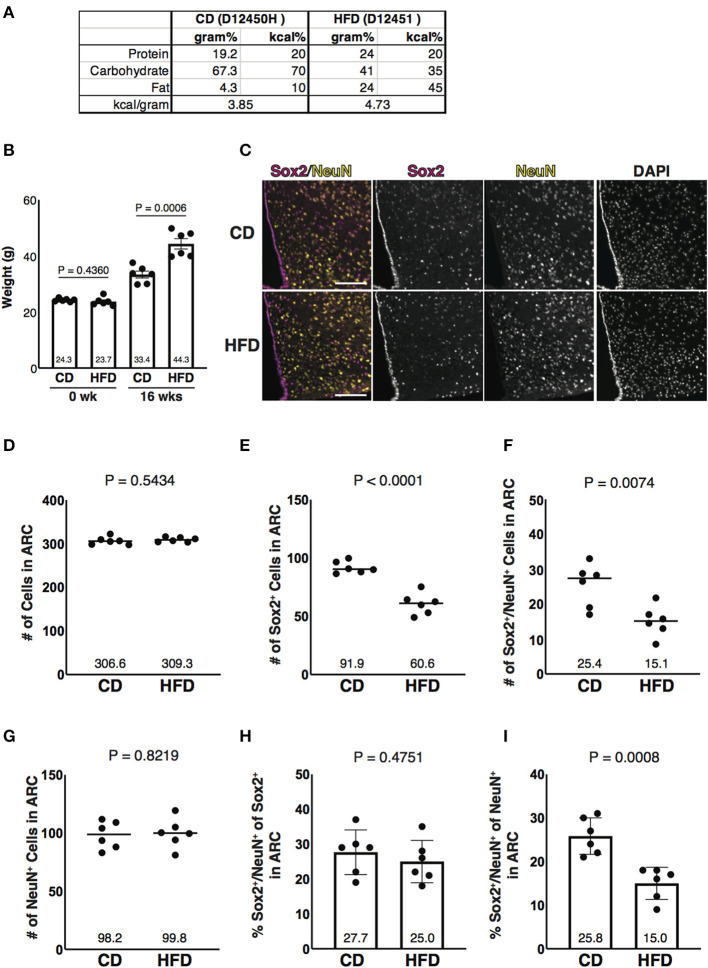
Diet-induced obesity-associated reduction of Sox2 and NeuN double-positive cells in number in the ARC. **(A)** Formula of diets used in this study. CD, control diet; HFD, high-fat diet. Both diets, as indicated in the catalog number, were purchased from Research Diets. **(B)** Bar graph represents the weight of animals before feeding CD or HFD (0 week) and after 16 weeks of CD or HFD feeding (16 weeks). The mean of each condition is indicated on graph. Error bars, SEM. **(C)** Representative images of the immunofluorescence labeling of Sox2 and NeuN and nuclear labeling with DAPI on the coronal sections of the ARC from mice fed with CD and HFD. Scale bars, 100 μm. **(D–G)** Reduction of Sox2 and NeuN double-positive cells, but not NeuN-positive cells, in number in HFD sample. Dot plots represent the number of all, Sox2-positive, Sox2 and NeuN double-positive, and NeuN-positive cells: **(D)** total number of cells counted by DAPI, **(E)** Sox2-positive cells, **(F)** Sox2 and NeuN double-positive cells, and **(G)** NeuN-positive cells. The mean of each condition is indicated on graphs and shown as horizontal lines. **(H,I)** Reduction of the ratio of Sox2 and NeuN double-positive cells to NeuN-positive cells, but not to Sox2-positive cells, in number in HFD sample. Bar graphs represent the ratio of Sox2 and NeuN double-positive cells to Sox2-positive and NeuN-positive cells: **(H)** Sox2-positive cells and **(I)** NeuN-positive cells. Error bars, SEM.

Obesity is an important risk factor for metabolic diseases that increase mortality risk. In diet-induced obese (DIO) mice, the loss of NSCs and the enhancement of inflammation have been observed in the ARC (Milanski et al., 2009; Li et al., 2012). These phenotypes are also found in aged mice, suggesting that obesity promotes hypothalamic aging (Li et al., 2012; Zhang et al., 2017). To test whether aging-associated reduction of Sox2 and NeuN double-positive cells in number in the parenchyma of the ARC is also found in DIO mice, mice were fed with HFD (Figure 4). After 16 weeks of feeding, HFD- and CD-fed animals gained weight from 23.7 to 44.3 g (1.87 folds, an average of six animals) and from 24.3 to 33.4 g (1.38 folds, an average of six animals), respectively (Figures 4A,B). As observed in aged mice, Sox2-positive cells, but not NeuN-positive cells, were reduced in number in HFD-fed mice (Figures 4C–G). Furthermore, the fraction of Sox2 and NeuN double-positive cells in NeuN-positive cells was significantly reduced in the ARC in the mice fed with HFD (Figures 4H,I). These results indicate that the reduced expression of Sox2 in the hypothalamic neuronal cells in the ARC is also induced by feeding HFD, which is known to accelerate aging.

## Discussion

Nestin and Sox2 double-positive tanycytes are widely accepted as the NSCs in the ARC of hypothalamus and are reduced in number by aging (Zhang et al., 2017). Nestin-negative Sox2-positive cells were previously observed in the parenchyma of the ARC (Li et al., 2012) but have not been characterized. We first confirmed here the presence of Nestin and Sox2 double-positive tanycytes and Nestin-negative Sox2-positive cells in the parenchyma of the ARC. We then found a part of Sox2-positive cells in the parenchyma of the ARC expressed NeuN. Quantitative analysis showed that these Sox2 and NeuN double-positive cells were reduced in number in aged mice, although DAPI-positive or NeuN-positive cells in the ARC were not reduced in each total number with aging. The Sox2 and NeuN double-positive cells in the ARC may be either terminally differentiated neurons with specific functions or neuronal cells in specific stages of the differentiation process that may or may not have their own specific functions. In the ARC, neuropeptide-releasing neurons, such as POMC- or NPY-expressing neurons, regulate food intake and energy expenditure (Cowley et al., 2001; Williams and Elmquist, 2012), and those neurons have been considered terminally differentiated neurons, but it has been reported that the expression of POMC is also observed in immature neurons (Padilla et al., 2010). In addition, it has also been shown that Sox2 regulates not only the maintenance of stemness but also neuronal functions (Mercurio et al., 2019b). Collectively, accumulated evidence indicates that terminally differentiated neurons and neuronal cells in the specific stages of the differentiation process cannot be distinguished only based on the expression of those functional molecules. We showed that Sox2 was expressed in a part of ACTH-positive cells but rarely in AgRP-positive cells. However, it cannot be concluded for these reasons whether Sox2 and NeuN double-positive cells in the ARC are terminally differentiated neurons with specific functions or neuronal cells in specific stages of the differentiation process. If Sox2 in the ARC neuronal cells possesses specific functions, it may play a role in homeostasis by regulating neuronal functions, and its reduction may lead to aging. Further characterization of Sox2 and NeuN double-positive cells in the ARC is required to address these issues.

It has been shown that neuronal death is induced by aging in the brain, particularly in the hippocampus and the cerebral cortex (Baker and Petersen, 2018). The aging-induced neuronal cell death is enhanced by stresses, such as metabolic stresses, hypoxia, and inflammation (Mattson and Arumugam, 2018). On the other hand, it has been shown that neuronal death is not induced by aging (Rapp and Gallagher, 1996). These controversial results may be dependent on different experimental conditions and brain regions (Markham et al., 2007). Thus, it remains to be established whether neuronal death is induced by aging in the brain, but it is evident that at least neuronal degeneration is induced by aging in the brain (Hou et al., 2019). As reported in rhesus monkey (Roberts et al., 2012), we showed that DAPI-positive or NeuN-positive cells in the ARC were not reduced in total number by aging or feeding HFD, indicating that neuronal cell death in the ARC is not induced by aging or feeding HFD. Therefore, the downregulation of Sox2 in Sox2 and NeuN double-positive cells may be caused by aging-associated degeneration by an unknown mechanism(s). The functions of Sox2 and NeuN double-positive cells in the ARC remain unresolved, but the reduction of Sox2 and NeuN double-positive cells in number in the ARC can be utilized as an aging marker for neuronal cells.

The functions of Sox2 have been investigated not only in stem and progenitor cells but also in differentiated neuronal cells. Dorsolateral geniculate nucleus plays a role in the visual pathway. Dorsolateral geniculate nucleus neuron-specific *Sox2*-knockout mice display defective visual due to the downregulation of serotonin transporter and neuronal projections (Chou et al., 2013; Mercurio et al., 2019a). The suprachiasmatic nucleus (SCN) plays a central role in controlling circadian rhythm by receiving visual information (Hâkansson et al., 1998). Deletion of *Sox2* in the SCN neuronal cells reduces Period 2 and neuropeptides release, leading to abnormal behavior (Cheng et al., 2019). Thus, Sox2 not only regulates the maintenance of stemness but also neuronal functions. We observed that Sox2 was expressed in the neuropeptide-releasing cells, suggesting that Sox2 regulates the release of these neuropeptides in the ARC. Indeed, NPY and POMC are linked to aging: NPY declines with aging (Gruenewald et al., 1994) and the NPY transgenic rats present longer life span (Michalkiewicz et al., 2003), whereas caloric restriction fails to extend the life span of NPY-deficient mice (Chiba et al., 2014). Furthermore, the elevation of K_ATP_ channel activity induced by increased mTOR signaling leads to hypertrophy of POMC neuronal cells, eventually developing aging-dependent obesity (Yang et al., 2012). Since tight junctions are incomplete in their barrier activity in tanycytes aligning the wall of the 3V in the ARC, its neuronal cells are exposed to CSF unlike neuronal cells in other regions of the hypothalamus where tight junctions of tanycytes have complete barrier activity to prevent an exposure to CSF (Mullier et al., 2010). Sox2 plays a role in anti-oxidative stress (Gopal et al., 2016). Neuronal cells may become vulnerable to stresses by the aging-associated reduction of Sox2. Further experiments using ARC neuronal cell-specific conditional Sox2 knockout mice are required to reveal the function of Sox2 in the Sox2 and NeuN double-positive cells in appetite/satiety and in aging.

During neuronal differentiation, the expression of Sox2 is downregulated post-transcriptionally and/or post-translationally (Pevny and Nicolis, 2010). MicroRNA-145 is known to suppress the transcription factors required for the maintenance of embryonic stem cells, such as Sox2, Oct4, and Klf4 (Xu et al., 2009). MicroRNA-145 also regulates neuronal differentiation by downregulating Sox2. An E3 ubiquitin ligase complex CUL4a^DET−COP1^ promotes the degradation of Sox2 *via* the ubiquitin-proteasome system, shifting NSCs/neural progenitor cells to neuronal cells (Cui et al., 2018). However, it is largely unknown whether microRNA-145 and CUL4a^DET−COP1^ are upregulated in aged neuronal cells. Importantly, the aging-associated reduction of proteasome activity is widely demonstrated in various organisms (Powers et al., 2009), suggesting that Sox2 is downregulated mechanistically rather than by aging-associated proteasomal dysfunction. Further studies are required to identify the upstream regulator of the aging-associated reduction of Sox2.

We showed that Sox2 was reduced in HFD-fed mice, a DIO model (Figure 4). Metabolic stresses, such as the elevation of fatty acids and oxidative stress, may be involved in the downregulation of Sox2. For example, the expression of Sox2 is also regulated by cyclin-dependent kinase inhibitor p21, which is upregulated under the oxidative stress (Marqués-Torrejón et al., 2013). It is interesting to analyze whether caloric restriction cancels or delays the reduction of Sox2. Furthermore, it is important to examine if the expression of Sox2 retained in the life span of mice, such as transgenic mice, proves the contribution of Sox2 to aging. Understanding the precise machinery underlying the aging-associated reduction of Sox2 in number in the hypothalamic neuronal cells will gain a new insight into preventing and/or retarding aging.

## Data Availability Statement

The original contributions presented in the study are included in the article, further inquiries can be directed to the corresponding author/s.

## Ethics Statement

The animal study was reviewed and approved by Kobe University Animal Care and Use Committee (approval number: 30-27).

## Author Contributions

KM and YT conceived the research project. AS designed the experiments. AS and TS performed the experiments. YT, KM, AS, TS, TK, TM, SK, and MM analyzed the data and wrote the manuscript. All authors contributed to the article and approved the submitted version.

## Conflict of Interest

The authors declare that the research was conducted in the absence of any commercial or financial relationships that could be construed as a potential conflict of interest.

## Abbreviations

ARC: arcuate nucleus
NSCs: neural stem cells
POMC: pro-opiomelanocortin
NPY: neuropeptide Y
AgRP: agouti-related protein
ACTH: adrenocorticotropic hormone
α-MSH: α-melanocyte-stimulating hormone
CSF: cerebrospinal fluid
HFD: high-fat diet
PB: phosphate buffer
Ab: antibody
PBS: phosphate-buffered saline
DAPI: 4′,6-diamidino-2-phenylindole
GFAP: glial fibrillary acidic protein
PDGFRα: platelet-derived growth factor receptor α
Iba1: ionized calcium-binding adapter molecule 1
DIO: diet-induced obese
SCN: suprachiasmatic nucleus.
